# Challenges to Ego-Depletion Research Go beyond the Replication Crisis: A Need for Tackling the Conceptual Crisis

**DOI:** 10.3389/fpsyg.2017.00568

**Published:** 2017-04-18

**Authors:** John H. Lurquin, Akira Miyake

**Affiliations:** Department of Psychology and Neuroscience, University of Colorado BoulderBoulder, CO, USA

**Keywords:** ego-depletion, self-control, the strength model, resource theories, replication crisis

One important line of self-control research concerns the phenomenon known as *ego-depletion*, the negative effect of performing a self-control task (Task 1) on performance on a subsequent self-control task (Task 2). Although a 2010 meta-analysis reported a moderate effect size (*d* = 0.62) for this phenomenon (Hagger et al., 2010), its replicability has since come under scrutiny with the publication of some replication failures (Xu et al., 2014; Lurquin et al., 2016), including a high-profile study involving 23 laboratories (Hagger et al., 2016). Some researchers even suggest that the ego-depletion effect might not be real and that the reported results primarily reflect publication bias (Carter and McCullough, 2014). This replication crisis has prompted a call for additional replication attempts involving large sample sizes and preregistration (Carter et al., 2015).

Although such replication efforts are undoubtedly important, we submit that, unless some fundamental conceptual (and related methodological) issues are more satisfactorily addressed, attempts to evaluate the ego-depletion effect would unlikely be successful. In this article, we outline what we call *the conceptual crisis* for the ego-depletion literature, explain how these limitations undermine replication attempts, and suggest possible ways to alleviate these problems. We do so by noting some parallel problems that have faced cognitive psychologists studying attention, working memory (WM), and executive functions (EFs), in the hope that such insights might contribute to theoretical and empirical development in ego-depletion research.

## The conceptual crisis surrounding the ego-depletion effect

We propose that compellingly resolving the controversy surrounding the ego-depletion effect requires concerted efforts to address three interrelated conceptual problems, which jointly make it difficult to derive unequivocal and testable predictions for any ego-depletion study. Below, we illustrate these problems by referring to the strength model of self-control (Baumeister et al., 2007) because this influential model has provided the basis for most of the existing ego-depletion research. We emphasize, however, that these problems are general enough to be also applicable to other models (e.g., Inzlicht and Schmeichel, 2012) and, hence, that field-wide efforts are needed to satisfactorily address them.

## 1. Lack of clear operational definitions of self-control

### Problem

*The field lacks clearly articulated and generally agreed-upon operational definitions of self-control that can guide ego-depletion research*. Although some studies refer to inhibitory control of some sort as their operational definition (e.g., Muraven et al., 2006; Tice et al., 2007), the term “inhibition” is typically used in a rather generic sense, without being specific as to different types of inhibitory processes postulated in the EF literature (Nigg, 2000; Friedman and Miyake, 2004). More problematic, some studies define self-control too broadly as the ability to control thoughts, emotions, and behavior (Segerstrom and Nes, 2007) or any monitoring and modification of behavior (Vohs et al., 2005).

The justifications used for selecting self-control tasks are equally unsatisfactory: Many studies use circular logic to justify task selection by noting that the task was used before and had a depleting effect. Even when some independent justifications are provided, the attributes used to justify a task vary greatly, including being intellectually demanding (Fennis et al., 2009), requiring effort (Boucher and Kofos, 2012), and simply being difficult (Webb and Sheeran, 2003). Consequently, wide-ranging tasks like taking standardized tests (e.g., Converse and Deshon, 2009) or even balancing on one leg (Tyler and Burns, 2008) count as self-control tasks.

Given this confusing state, it is hardly surprising that the same task (e.g., 3-digit by 3-digit multiplication) has been used as both the self-control (depletion) task (Stillman et al., 2009) and the control (nondepletion) task (Burkley, 2008). If one cannot unambiguously determine whether or not a particular task implicates self-control, it is impossible to determine whether one should expect a significant ego-depletion effect.

### Ways forward

Each researcher should explicitly articulate an operational definition of self-control used in his/her study and justify task selection with regard to that operational definition. To facilitate the progress, however, more needs to be done by the field as a whole. Parallel conceptual problems that have faced EF research—another elusive and multifaceted concept—may be relevant here. Although it is still far from achieving a field-wide consensus (Baggetta and Alexander, 2016), attempts to systematically classify and operationally define different facets of EFs (e.g., updating, shifting, and inhibition; Miyake et al., 2000) have contributed to developing some initial consensus, which has helped researchers judge whether a task implicates EF processes. Analogous attempts would be helpful for self-control research, especially if such efforts can help systematically examine which facets of self-control are linked to the ego-depletion phenomenon (e.g., Fujita, 2011; Heller et al., 2017).

## 2. Lack of independent empirical validation for self-control tasks

### Problem

*Various tasks used in ego-depletion research*—such as watching a video while ignoring words appearing onscreen and writing essays without using certain letters—*have not been independently validated as effective measures of self-control*. Some such tasks have not been used outside ego-depletion research, and some (e.g., the video-viewing task) even lack objective measures of task performance that could be used as indices of self-control.

This lack of independent validation of self-control tasks is problematic, because it makes it difficult to derive an unambiguous prediction for any ego-depletion study. For example, according to the strength model, the ego-depletion effect should be observed only when Tasks 1 and 2 (a) both implicate self-control and (b) draw from the same self-control resources. It is unclear, however, whether various task combinations used in ego-depletion research actually meet these necessary conditions.

Concerning (a), a negative consequence of this problem is illustrated by recent exchanges (Baumeister and Vohs, 2016b; Hagger and Chatzisarantis, 2016) regarding the appropriateness, as a self-control task, of the specific *e*-crossing task used in Hagger et al. (2016) multilab replication study. A focal issue was the necessity of an initial habit-forming block to make the *e*-crossing task sufficiently demanding, but, tellingly, this exchange did not reference any independent (non-ego-depletion) research validating different versions of the *e*-crossing task as effective (or not-so-effective) indices of self-control. Without such independent evidence, any replication failures would be open for alternative explanations based on task-selection problems.

Concerning (b), we do not know of any independent evidence for this crucial domain-generality assumption. Although there has been rigorous theoretical debate about, and empirical investigation into, the domain generality/specificity of attention (e.g., Wickens, 1984) and WM (e.g., Kane et al., 2004), little consideration has been given to this important issue in ego-depletion research, despite some prior evidence for domain/process-specific ego-depletion effects (Persson et al., 2007; Healey et al., 2011). Moreover, this domain-generality assumption is built on circular logic: Domain-general self-control resources must be present because the ego-depletion effect is observed. This criticism is reminiscent of those raised against resource theories in cognitive psychology, most notably Kahneman's (1973) seminal capacity theory of attention, which, like the strength model, postulated a single pool of general-purpose attentional resources fueling various mental activities.^1^

We find it justifiable to initially develop laboratory self-control tasks on the basis of the experimenter's intuition (Baumeister, 2016). We expect, however, that subsequent research would validate their appropriateness as self-control indicators and offer independent evidence that these tasks indeed draw on the same pool of domain-general self-control resources. Without knowing whether a particular task combination used in a study meets these conditions, it is impossible to predict whether one should expect a significant ego-depletion effect in that study.

### Ways forward

One way to alleviate these problems is to conduct carefully designed correlational research (e.g., latent-variable analysis) and/or experimental studies using the simultaneous dual-task interference paradigm to establish that various commonly used tasks in ego-depletion research share some underlying commonality, namely self-control resources. Tests of ego-depletion would be more effective when the specific combination of tasks used has already been shown to demonstrate a clear overlap between them. In this regard, relying more on cognitive (attention, WM, and EF) tasks for which such evidence of overlap already exists might be helpful.

It is also important to provide more objective measures of task performance to quantify the self-control demands associated with Task 1 performance. One such possibility is to use pupillometry (Beatty, 1982) as an index of the degree of *effort* or attentional demands associated with the task performance^2^ (e.g., Hopstaken et al., 2015; Rondeel et al., 2015).

## 3. Lack of well-specified models that make unambiguous, falsifiable predictions

### Problem

*The existing models purported to explain the ego-depletion effect are currently too underspecified to allow other researchers to unambiguously derive testable (falsifiable) predictions*. For example, the strength model does not specify how the self-control resources are consumed by Tasks 1 and 2 and when the available remaining resources are low enough to start impairing subsequent performance on Task 2. Such key resource-consumption parameters must be more formally specified before one can determine whether an experiment should produce the ego-depletion effect.

This theoretical issue has been neglected in ego-depletion research, despite some relevant historical precedent. In an influential critique, Navon (1984) articulated various problems with resource theories (e.g., Kahneman, 1973), including the aforementioned circularity problem and the ambiguity surrounding the hypothesized resource-performance functions. This critique led some theorists to abandon the resource concept altogether (Neuman, 1987) and others to attempt to better specify the nature of resources and their consumption functions in the form of computational models (e.g., Just and Carpenter, 1992; Lovett et al., 1999). Models of ego-depletion phenomena are in need of such formalization.

Such theoretical development is urgently needed following the recent updates made to the strength model (Baumeister and Vohs, 2016a) that, in our view, make the model flexible enough to fit any data and, hence, unfalsifiable. In particular, this revised model incorporates the notion of the “central governor” (adopted from Evans et al., 2016), whose role is to determine whether to expend or conserve the available self-control resources. This addition seems to us a step backwards, considering that WM theories, which have long featured the “central executive” (Baddeley and Hitch, 1974; Baddeley, 1996), have been trying to replace this vague, homunculus-like construct with something more precise^3^. Without better specifying how this central governor determines whether and when to consume or conserve self-control resources, one cannot unambiguously determine whether one should observe a significant ego-depletion effect (for a more detailed critique of the central governor model, see Inzlicht and Marcora (2016).

### Ways forward

We do not know of any formal attempts to mechanically specify how self-control resources are consumed when two tasks are performed consecutively in the sequential-task paradigm. It seems necessary not only to better specify the underlying resource-consumption functions (preferably via mathematical or computational modeling) but also to be more explicit about critical moderating variables (e.g., when to conserve or consume resources). To gain insights into the underlying resource-performance functions, it might also be helpful to systematically (parametrically) manipulate task durations or attentional demands for Task 1 (Lee et al., 2016), which unfortunately has rarely been done in ego-depletion research.

## Conclusion

The recent replication efforts have succeeded in promoting preregistration, open data, and large sample sizes, all of which improve the reproducibility of scientific work. To resolve the issue of whether ego-depletion is a real phenomenon, however, it is also crucial to address the severe conceptual problems that impede the derivation and testing of specific, falsifiable predictions. Although tackling these issues is not easy, we believe that effectively addressing them is a necessary step to resolve the current controversy surrounding the ego-depletion effect in a manner that satisfies its proponents and skeptics alike.

## Author contributions

All authors listed, have made substantial, direct and intellectual contribution to the work, and approved it for publication.

## Funding

Publication of this article was funded by the University of Colorado Boulder Libraries Open Access Fund.

### Conflict of interest statement

The authors declare that the research was conducted in the absence of any commercial or financial relationships that could be construed as a potential conflict of interest.

## Footnotes

^1^If the glucose-as-self-control-resources hypothesis (Gailliot et al., 2007) had received strong support, this would have resolved the circular-logic problem, but, given the growing evidence against this hypothesis (Beedie and Lane, 2012; Dang, 2016; Vadillo et al., 2016), the field still lacks independent, noncircular evidence for the domain-generality assumption.

^2^If *fatigue* is the more critical dimension (Baumeister and Vohs, 2016b; Drummond and Philipp, 2017), independent evidence for linking fatigue levels to self-control resources is needed.

^3^Indeed, Logie (2016), a long-time proponent of the central executive (Baddeley and Logie, 1999), recently declared that the field is now ready to “retire” it.

## References

[B1] Baddeley A. (1996). Exploring the central executive. Q. J. Exp. Psychol. 49A, 5–28. 10.1080/713755608

[B2] Baddeley A. D. Hitch G. J. (1974). Working memory, in The Psychology of Learning and Motivation, ed Bower G. H. (New York, NY: Academic Press), 47–89.

[B3] Baddeley A. D. Logie R. H. (1999). Working memory: the multiple-component model, in Models of Working Memory: Mechanisms of Active Maintenance and Executive Control, eds Miyake A. Shah P. (Cambridge: Cambridge University Press), 28–61.

[B4] Baggetta P. Alexander P. A. (2016). Conceptualization and operationalization of executive function. Mind Brain Educ. 10, 10–33. 10.1111/mbe.12100

[B5] Baumeister R. F. (2016). Charting the future of social psychology on stormy seas: winners, losers, and recommendations. J. Exp. Soc. Psychol. 66, 153–158. 10.1016/j.jesp.2016.02.003

[B6] Baumeister R. F. Vohs K. D. (2016a). Strength model of self-regulation as limited resource: assessment, controversies, update. Adv. Exp. Soc. Psychol. 54, 67–127. 10.1016/bs.aesp.2016.04.001

[B7] Baumeister R. F. Vohs K. D. (2016b). Misguided effort with elusive implications. Perspect. Psychol. Sci. 11, 574–575. 10.1177/1745691616652878 27474143

[B8] Baumeister R. F. Vohs K. D. Tice D. M. (2007). The strength model of self-control. Curr. Dir. Psychol. Sci. 16, 351–355. 10.1111/j.1467-8721.2007.00534.x

[B9] Beatty J. (1982). Task-evoked pupillary responses, processing load, and the structure of processing resources. Psychol. Bull. 91, 276–292. 10.1037/0033-2909.91.2.276 7071262

[B10] Beedie C. J. Lane A. M. (2012). The role of glucose in self-control: another look at the evidence and an alternative conceptualization. Pers. Soc. Psychol. Rev. 16, 143–153. 10.1177/1088868311419817 21896791

[B11] Boucher H. C. Kofos M. N. (2012). The idea of money counteracts ego depletion effects. J. Exp. Soc. Psychol. 48, 804–810. 10.1016/j.jesp.2012.02.003

[B12] Burkley E. (2008). The role of self-control in resistance to persuasion. Pers. Soc. Psychol. Bull. 34, 419–431. 10.1177/0146167207310458 18272808

[B13] Carter E. C. Kofler L. M. Forster D. E. McCullough M. E. (2015). A series of meta-analytic tests of the depletion effect: self-control does not seem to rely on a limited resource. J. Exp. Psychol. 144, 796–815. 10.1037/xge0000083 26076043

[B14] Carter E. C. McCullough M. E. (2014). Publication bias and the limited strength model of self-control: has the evidence for ego depletion been overestimated? Front. Psychol. 5:823. 10.3389/fpsyg.2014.00823 25126083 PMC4115664

[B15] Converse P. D. Deshon R. P. (2009). A tale of two tasks: reversing the self-regulatory resource depletion effect. J. Appl. Psychol. 94, 1318–1324. 10.1037/a0014604 19702373

[B16] Dang J. (2016). Testing the role of glucose in self-control: a meta-analysis. Appetite 107, 222–230. 10.1016/j.appet.2016.07.021 27492453

[B17] Drummond A. Philipp M. C. (2017). Commentary: “Misguided effort with elusive implications” and “A multi-lab pre-registered replication of the ego depletion effect.” Front. Psychol. 8:273. 10.3389/fpsyg.2017.00273 28293205 PMC5329006

[B18] Evans D. R. Boggero I. A. Segerstrom S. C. (2016). The nature of self-regulatory fatigue and “ego-depletion”: lessons from physical fatigue. Pers. Soc. Psychol. Rev. 20, 291–310. 10.1177/1088868315597841 PMC4788579 26228914

[B19] Fennis B. M. Janssen L. Vohs K. D. (2009). Acts of benevolence: a limited resource account of compliance with charitable requests. J. Consum. Res. 35, 906–924. 10.1086/593291

[B20] Friedman N. P. Miyake A. (2004). The relations among inhibition and interference control functions: a latent-variable analysis. J. Exp. Psychol. 133, 101–135. 10.1037/0096-3445.133.1.101 14979754

[B21] Fujita K. (2011). On conceptualizing self-control as more than the effortful inhibition of impulses. Pers. Soc. Psychol. Rev. 15, 352–366. 10.1177/1088868311411165 21685152

[B22] Gailliot M. T. Baumeister R. F. DeWall C. N. Maner J. K. Plant E. A. Tice D. M.. (2007). Self-control relies on glucose as a limited energy source: willpower is more than a metaphor. J. Pers. Soc. Psychol. 92, 325–336. 10.1037/0022-3514.92.2.325 17279852

[B23] Hagger M. S. Chatzisarantis N. L. (2016). Commentary: misguided effort with elusive implications, and sifting signal from noise with replication science. Front. Psychol. 7:621. 10.3389/fpsyg.2016.00621 27561304 PMC4987495

[B24] Hagger M. S. Chatzisarantis N. L. Alberts H. A. Anggono C. O. Batailler C. B. Birt A. R.. (2016). A multilab preregistered replication of the ego-depletion effect. Pers. Psychol. Sci. 11, 546–573. 10.1177/1745691616652873 27474142

[B25] Hagger M. S. Wood C. Stiff C. Chatzisarantis N. L. (2010). Ego depletion and the strength model of self-control: a meta-analysis. Psychol. Bull. 136, 495–525. 10.1037/a0019486 20565167

[B26] Healey M. K. Hasher L. Danilova E. (2011). The stability of working memory: do previous tasks influence complex span? J. Exp. Psychol. 140, 573–585. 10.1037/a0024587 21767041

[B27] Heller S. Borsay F. Ullrich J. (2017). Social power and dimensions of self-control: does power benefit initiatory self-control but impair inhibitory self-control? Cogent Psychol. 4:1288351 10.1080/23311908.2017.1288351

[B28] Hopstaken J. F. van der Linden D. Bakker A. B. Kompier M. A. (2015). The window of my eyes: task disengagement and mental fatigue covary with pupil dynamics. Biol. Psychol. 110, 100–106. 10.1016/j.biopsycho.2015.06.013 26196899

[B29] Inzlicht M. Marcora S. M. (2016). The central governor model of exercise regulation teaches us precious little about the nature of mental fatigue and self-control failure. Front. Psychol. 7:656. 10.3389/fpsyg.2016.00656 27199874 PMC4854881

[B30] Inzlicht M. Schmeichel B. J. (2012). What is ego depletion? Toward a mechanistic revision of the resource model of self-control. Pers. Psychol. Sci. 7, 450–463. 10.1177/1745691612454134 26168503

[B31] Just M. A. Carpenter P. A. (1992). A capacity theory of comprehension: individual differences in working memory. Psychol. Rev. 99, 122–149. 10.1037/0033-295x.99.1.122 1546114

[B32] Kahneman D. (1973). Attention and Effort. Englewood Cliffs, NJ: Prentice-Hall.

[B33] Kane M. J. Hambrick D. Z. Tuholski S. W. Wilhelm O. Payne T. W. Engle R. W. (2004). The generality of working memory capacity: a latent-variable approach to verbal and visuospatial memory span and reasoning. J. Exp. Psychol. 133, 189–217. 10.1037/0096-3445.133.2.189 15149250

[B34] Lee N. Chatzisarantis N. Hagger M. S. (2016). Adequacy of the sequential-task paradigm in evoking ego-depletion and how to improve detection of ego-depletion phenomena. Front. Psychol. 7:136. 10.3389/fpsyg.2016.00136 26903932 PMC4746433

[B35] Logie R. H. (2016). Retiring the central executive. Q. J. Exp. Psychol. 69, 2093–2109. 10.1080/17470218.2015.1136657 26821744

[B36] Lovett M. C. Reder L. M. Lebiere C. (1999). Modeling working memory in a unified architecture: an ACT-R perspective, in Models of Working Memory: Mechanisms of Active Maintenance and Executive Control, eds Miyake A. Shah P. (Cambridge: Cambridge University Press), 135–182.

[B37] Lurquin J. H. Michaelson L. E. Barker J. E. Gustavson D. E. von Bastian C. C. Carruth N. P.. (2016). No evidence of the ego-depletion effect across task characteristics and individual differences: a pre-registered study. PLoS ONE 11:e0147770. 10.1371/journal.pone.0147770 26863227 PMC4749338

[B38] Miyake A. Friedman N. P. Emerson M. J. Witzki A. H. Howerter A. Wager T. D. (2000). The unity and diversity of executive functions and their contributions to complex “frontal lobe” tasks: a latent variable analysis. Cogn. Psychol. 41, 49–100. 10.1006/cogp.1999.0734 10945922

[B39] Muraven M. Shmueli D. Burkley E. (2006). Conserving self-control strength. J. Pers. Soc. Psychol. 91, 524–537. 10.1037/0022-3514.91.3.524 16938035

[B40] Navon D. (1984). Resources—a theoretical soup stone? Psychol. Rev. 91, 216–234. 10.1037/0033-295X.91.2.216

[B41] Neuman O. (1987). Beyond capacity: a functional view of attention, in Perspectives on Selection and Action, eds Heuer H. Sanders A. F. (Hillsdale, NJ: Erlbaum), 361–394.

[B42] Nigg J. T. (2000). On inhibition/disinhibition in developmental psychopathology: views from cognitive and personality psychology and a working inhibition taxonomy. Psychol. Bull. 126, 220–246. 10.1037/0033-2909.126.2.220 10748641

[B43] Persson J. Welsh K. M. Jonides J. Reuter-Lorenze P. A. (2007). Cognitive fatigue of executive processes: interaction between interference resolution tasks. Neuropsychologia 45, 1571–1579. 10.1016/j.neuropsychologia.2006.12.007 17227678 PMC1876692

[B44] Rondeel E. W. van Steenbergen H. Holland R. W. van Knippenberg A. (2015). A closer look at cognitive control: differences in resource allocation during updating, inhibition and switching as revealed by pupillometry. Front. Hum. Neurosci. 9:494. 10.3389/fnhum.2015.00494 26441594 PMC4564574

[B45] Segerstrom S. C. Nes L. S. (2007). Heart rate variability reflects self-regulatory strength, effort, and fatigue. Psychol. Sci. 18, 275–282. 10.1111/j.1467-9280.2007.01888.x 17444926

[B46] Stillman T. F. Tice D. M. Fincham F. D. Lambert N. M. (2009). The psychological presence of family improves self-control. J. Soc. Clin. Psychol. 28, 498–529. 10.1521/jscp.2009.28.4.498

[B47] Tice D. M. Baumeister R. F. Shmueli D. Muraven M. (2007). Restoring the self: positive affect helps improve self-regulation following ego depletion. J. Exp. Soc. Psychol. 43, 379–384. 10.1016/j.jesp.2006.05.007

[B48] Tyler J. M. Burns K. C. (2008). After depletion: the replenishment of the self's regulatory resources. Self Identity 7, 305–321. 10.1080/15298860701799997

[B49] Vadillo M. A. Gold N. Osman M. (2016). The bitter truth about sugar and willpower: the limited evidential value of the glucose model of ego depletion. Psychol. Sci. 27, 1207–1214. 10.1177/0956797616654911 27485134

[B50] Vohs K. D. Baumeister R. F. Ciarocco N. J. (2005). Self-regulation and self-presentation: regulatory resource depletion impairs impression management and effortful self-presentation depletes regulatory resources. J. Pers. Soc. Psychol. 88, 632–657. 10.1037/0022-3514.88.4.632 15796665

[B51] Webb T. L. Sheeran P. (2003). Can implementation intentions help to overcome ego-depletion? J. Exp. Soc. Psychol. 39, 279–286. 10.1016/S0022-1031(02)00527-9

[B52] Wickens C. D. (1984). Processing resources in attention, in Varieties of Attention, eds Parasuraman R. Davies D. R. (Orlando, FL: Academic Press), 63–102.

[B53] Xu X. Demos K. E. Leahey T. M. Hart C. N. Trautvetter J. Coward P.. (2014). Failure to replicate depletion of self-control. PLoS ONE 9:e109950. 10.1371/journal.pone.0109950 25333564 PMC4204816

